# Valorization of *Aloe vera* Skin By-Products to Obtain Bioactive Compounds by Microwave-Assisted Extraction: Antioxidant Activity and Chemical Composition

**DOI:** 10.3390/antiox11061058

**Published:** 2022-05-26

**Authors:** Ignacio Solaberrieta, Alfonso Jiménez, María Carmen Garrigós

**Affiliations:** Department of Analytical Chemistry, Nutrition & Food Sciences, University of Alicante, San Vicente del Raspeig, ES-03690 Alicante, Spain; solaberrieta@ua.es (I.S.); alfjimenez@ua.es (A.J.)

**Keywords:** *Aloe vera*, waste valorization, microwave-assisted extraction (MAE), antioxidant activity, phenolic profile, Box-Behnken design (BBD)

## Abstract

*Aloe vera* skin (AVS) is a major by-product of Aloe processing plants all over the world. In this study, response surface methodology was used to optimize microwave-assisted extraction (MAE) of bioactive compounds from AVS. The influence of extraction parameters, such as ethanol concentration (%Et), extraction temperature (T), time (t) and solvent volume (V), on extraction yield (Y), total phenolic content (TPC), antioxidant activity (DPPH and FRAP methods) and aloin content, was studied. Optimum extraction conditions were determined as 80% ethanol, 80 °C, 36.6 min and 50 mL and optimized extracts showed interesting contents of polyphenols and antioxidant performance. The phenolic profile was determined by HPLC-DAD/MS and some major phenolic compounds, such as aloin A, aloin B, aloesin, aloe-emodin, aloeresin D, orientin, cinnamic acid and chlorogenic acid, were quantified while eight other compounds were tentatively identified. Moreover, structural and thermal properties were studied by FTIR and TGA analyses, respectively. The obtained results suggested the potential of AVS as a promising source of bioactive compounds, thus increasing the added value of this agricultural waste.

## 1. Introduction

In recent decades, growing environmental concerns combined with the imminent depletion of fossil fuels have encouraged the concept of the circular economy as an environmentally friendly approach to prevent waste generation in productive processes, as well as to develop natural-based products in order to reduce dependence on fuel-based materials [1]. The use of biopolymers or active biomolecules obtained from agricultural by-products or wastes has gained major importance in many countries around the world due to its environmental and economic advantages [2]. Recent studies have reported a wide variety of agrowastes as renewable sources of bioactive compounds or biopolymers that are currently underexploited, such as *Aloe vera* skin [3,4], cocoa bean shells [5], tomato seeds [6] and carob pod bark [7], among many others.

*Aloe vera* has been associated with curative or healing-promoting properties since ancient times. Several studies have demonstrated its anticancer, antiviral, anti-inflammatory, antimicrobial and antioxidant activities [8,9]. Pharmaceutical, food and cosmetic industries have used *Aloe vera* in innumerable product formulations which have been continuously growing in recent years [10,11]. The most valuable part of the plant is the inner leaf gel, which is used for product manufacturing. In *Aloe vera* processing plants, the inner gel is generally separated from the external skin, which accounts for over 30% of the total leaf weight, generating large amounts of waste. In most cases, this agrowaste lacks commercial use and is usually discarded, used as compost or directly incinerated [12]. Even though there is little information about *Aloe vera* skin compared to the inner gel, which has been extensively studied, it has been reported that *Aloe vera* skin could be a promising source of bioactive compounds which might be used in food, food packaging or biomedical applications [4,13,14].

There is a general consensus that biological activities of medicinal plants, including *Aloe vera*, which present very complex phytochemical profiles and many constituents, should be ascribed to the synergistic action of several compounds rather than to a single molecule. More than 75 active compounds have been identified in *Aloe vera*, with some of them, such as polysaccharides, related to wound healing, anti-inflammatory and antidiabetic activities [8,15]. However, the specific roles of other components have not been completely elucidated yet and some of them are suspected to be responsible for undesirable effects when consumed. This is the case with aloin, an anthraquinone-C-glycoside which is present, especially, in the latex of *Aloe vera* leaves and naturally occurs as a mixture of diastereoisomers, aloin A and aloin B [16]. Even though some studies have highlighted some protective benefits of anthraquinones, such as hepatoprotective, anticancer, antimicrobial and antioxidant activities [17,18,19], it has been reported to have a strong laxative effect, and it might cause other undesirable effects [20,21]. The maximum content of aloin in products intended for oral consumption is limited by specific regulations such as in North America, where the International Science Aloe Council has set a limit of 10 ppm of aloin A and B [20]. On the other hand, the European Council Directive 88/388/EEC established an aloin limit of 0.1 ppm in flavorings for beverages [22].

The extraction of bioactive molecules from plants could be carried out by using a variety of methods [23]. In recent years, several non-conventional and more environmentally friendly extraction techniques, which usually lead to higher overall extraction yields with less time and solvent consumption, have been developed [24,25]. Among them, microwave-assisted extraction (MAE) has gained importance due to its multiple advantages compared to conventional extraction techniques [26,27]. The synergistic combination of heat and mass transfer phenomena, in which both gradients work in the same direction, combined with volumetric heat dissipation inside the irradiated medium, are the most distinctive characteristics of MAE [28]. Moreover, it has been reported that this heating mechanism generates internal pressure into the plant materials, contributing to the rupture of cell walls and promoting solvent penetration into the vegetal matrix. Therefore, active compounds could be easily solubilized and extracted [28].

Most plant extracts comprise complex mixtures of different phytochemical constituents with diverse chemical structures, and their analysis remains challenging. Currently, several literature reviews on the identification and quantification of active compounds extracted from plants are available [29,30,31]. Even though some analytical techniques, such as gas chromatography and capillary electrophoresis, have been employed, high performance liquid chromatography (HPLC) combined with mass spectrometry (MS) has been by far the most frequently used technique for the identification and quantification of secondary metabolites extracted from plants [32,33,34,35]. Successful phenolic profiling strongly relies on proper optimization of chromatographic conditions, which are crucial for the selection of adequate mobile phases and gradient elution programs.

MAE has been used to extract bioactive compounds from several agrowastes [36]. However, to the best of our knowledge, no studies have been carried out for the MAE optimization of bioactive compounds with antioxidant activity from *Aloe vera* skin, adding value to this underexploited renewable resource. In this context, this work aimed at optimizing a new extraction procedure for bioactive compounds from *Aloe vera* skin in order to increase the added value of this agricultural waste. The optimal MAE conditions for maximizing the recovery of active phenolic compounds and the antioxidant activity of the extracts were determined. The influence of four extraction variables (irradiation time, extraction temperature, ethanol concentration in the extraction solvent and volume) was studied and the MAE process was optimized using a Box-Benhken design (BBD). Total phenolic content (TPC), antioxidant activity by DPPH and FRAP methods and aloin content in *Aloe vera* skin extracts were evaluated as the response variables. Moreover, phenolic profile, thermal and structural properties of the optimized *Aloe vera* skin extract were also determined by HPLC-DAD/MS, thermogravimetric analysis (TGA) and Fourier transform infrared spectroscopy (FTIR), respectively. Finally, morphological changes on the AVS structure due to the MAE process were studied by scanning electron microscopy (SEM).

## 2. Materials and Methods

### 2.1. Reagents and Raw Materials

Sodium carbonate, Folin-Ciocalteu reagent (2 N), 2,2-diphenyl-1-picrylhydrazyl (DPPH), sodium acetate, glacial acetic acid, hydrochloric acid, 2,4,6-tripyridyl-s-triazine (TPTZ), ferric chloride hexahydrate, gallic acid, 6-hydroxy-2,5,7,8-tetramethylchroman-2-carboxylic acid (Trolox), ethanol (absolute grade), methanol, acetonitrile (HPLC grade), formic acid and cinnamic acid were purchased from Sigma Aldrich (Madrid, Spain). Aloin A, aloin B, orientin, aloeresin D, aloesin and aloe-emodin were acquired from Cymit Química (Barcelona, Spain). Chlorogenic acid and all other phenolic standards used in this study were purchased from Extrasynthese (Genay, France). Ultrapure water from a Millipore Milli-Q system was used (18.2 MΩ·cm at 25 °C).

Fresh *Aloe vera* leaves, from three-year old plants, were kindly supplied by *Las Coronas* (Carnota, Seville, Spain), and they were thoroughly washed with distilled water to remove the adhering soil particles and other contaminants. Then, leaves were weighted and measured for their length, thickness and width (Table 1). Subsequently, the base, tip and spikes were removed, and the epidermis was carefully separated from the inner gel using a sharp knife. The yield of *Aloe vera* waste was determined and expressed as a fraction of the total leaf weight. The resulting *Aloe vera* skin was intensively washed with distilled water and the residual gel was removed. Then, it was cut into small pieces and frozen at −80 °C. Afterwards, the skin was freeze-dried with a Telstar Lyoquest—55 PLUS (Terrassa, Barcelona, Spain) and ground using a ZM 200 high-speed rotatory mill (Restch, Hann, Germany). Particles passing through a 1.0 mm sieve were used to ensure the homogeneity of the sample. Finally, the freeze-dried *Aloe vera* skin powder (AVS) was vacuum packed and stored in the darkness until further analysis.

### 2.2. AVS Characterization

AVS characterization was carried out by following AOAC official methods [37] and reported works for the analysis of similar plant materials [3,38]. Total content of lipids was gravimetrically determined after 8 h of Soxhlet extraction using petroleum ether. The crude protein content was calculated using the Kjeldahl method by multiplying the nitrogen value by 6.25. Total ash was determined by incineration in a muffle at 550 °C for 4 h. The moisture content was determined after skin separation by freeze-drying. All analyses were carried out in triplicate.

### 2.3. Microwave-Assisted Extraction (MAE)

MAE was performed using a Milestone flexiWAVE^TM^ microwave oven (Milestone srl, Bergamo, Italy) in the open vessel configuration, and the solvent was heated and refluxed through the sample. Ethanol:water mixtures were used as reported efficient solvents for the extraction of phenolic compounds from plant materials [7,39,40], with ethanol showing low toxicity [41]. According to preliminary tests, the AVS amount was fixed at 1.5 g. The initial heating rate was optimized and held constant among the different extraction experiments. During the extraction process, samples were stirred at 400 rpm and different combinations of solvent composition (%Et), extraction temperature (T), extraction time (t) and volume (V) were used according to Table 2. After extraction, the obtained *Aloe vera* skin extracts (AVE) were cooled to room temperature and centrifuged at 5300 rpm for 10 min. The solid residue was washed twice with the extraction solvent and then discarded. Then, the supernatant was pooled with the washing solvent and stored overnight at −20 °C in order to remove possible interferences by precipitation. After that, the precipitates were removed by centrifugation at 5300 rpm and 4 °C for 10 min. The supernatant was collected, and the ethanol was subsequently evaporated under reduced pressure. Afterwards, the extract was frozen at −80 °C, freeze-dried until completely dry and finally stored in vacuum-sealed packs at −20 °C in darkness until further analysis. AVE solutions were freshly prepared, before analysis, at 500 mg kg^−1^ in ethanol:water (40%, *v/v*).

#### Box-Behnken Experimental Design (BBD)

MAE optimization of bioactive compounds from AVS was performed by using a BBD with 29 runs and 5 central points in order to maximize extraction yield, total phenolic content (TPC) and antioxidant activity (DPPH and FRAP), as well as to minimize aloin content. The effect of four independent variables (ethanol concentration (%Et: 40–80%, *v/v*), temperature (T: 40–80 °C), time (t: 5–40 min) and solvent volume (V: 50–80 mL)) were investigated at three levels. All experimental runs were performed randomly to minimize the effect of unexpected variability in the response variables. The range of the studied independent variables was selected on the basis of preliminary experiments (data not shown), experimental limitations, constructive characteristics of the used equipment and previously reported information in the literature.

Response surface methodology (RSM) was used, and the following second-order polynomial model was applied for regression analysis of the experimental data:

(1) $$Y={\;\beta }_{0}+\sum^{\;}\beta_{i}X_{i}+\sum^{\;}\beta_{i}X_{i}^{2}+\sum^{\;}\sum^{\;}\beta_{\mathrm{ij}}X_{i}X_{j}$$

where Y represents the predicted response variable; X_i_ and X_j_ represent the independent variables; β_0_ is a constant coefficient and β_i_, β_ii_, β_ij_ are the regression coefficients of the linear, quadratic and interaction effect terms, respectively. The lack of fit test and coefficient of determination (R^2^) were used to determine the adequacy of the model to predict experimental data. Statistical significance of the model parameters was determined at 5% probability level (α = 0.05), and graphic analysis of the principal effects and interactions of independent variables on the studied responses was used.

### 2.4. AVE Characterization

#### 2.4.1. Extraction Yield

The extraction yield was gravimetrically determined by using the following equation:

(2) $$\mathrm{Yield}\;\left(\%\right)=100\;\frac{m_{\mathrm{AVE}}}{m_{\mathrm{AVS}}}$$

where m_AVE_ is the weight of the extract obtained after freeze-drying and m_AVS_ is the weight of the dried starting material.

#### 2.4.2. Total Phenolic Content (TPC)

The total phenolic content of AVE was determined using the Folin-Ciocalteu assay according to Lucini et al. [4] with slight modifications. Aliquots (0.5 mL) of each extract were mixed with 2.5 mL of the Folin-Ciocalteu reagent, previously diluted in distilled water (1:10, *v*/*v*), and added with 2.0 mL of 7.5 wt% aqueous sodium carbonate. Then, the mixture was vortexed, and the absorbance was recorded at 765 nm after 30 min of incubation at 45 °C in the dark using a Biomate 3 UV-vis spectrophotometer (Thermospectronic, Mobile, AL, USA). Gallic acid in EtOH:H_2_O (40%, *v*/*v*) was used as a reference standard for quantification (5–80 mg kg^−1^, R^2^ = 0.9995). The results were expressed as milligrams of gallic acid equivalents (GAE) per gram of AVE. Each extract was analyzed in triplicate.

#### 2.4.3. Antioxidant Activity

##### 2.4.3.1. DPPH Radical Scavenging Assay

The DPPH scavenging activity of AVE was determined, in triplicate, as described in previous studies [42,43,44] with some modifications. Briefly, 0.3 mL of AVE were mixed with 2.2 mL of a freshly prepared DPPH solution (10^−4^ mol L^−1^ in ethanol). The mixture was vortexed and incubated at room temperature in the dark for 90 min. Preliminary studies demonstrated that this amount of time was required to accomplish the reaction and reach the steady state. Then, absorbance was measured at 517 nm against a pure ethanol blank. Trolox in EtOH:H_2_O (40%, *v*/*v*) was used as the reference standard for quantification (5–70 mg kg^−1^, R^2^ = 0.9994). Results were expressed as milligrams of trolox equivalents (TE) per gram of AVE.

##### 2.4.3.2. FRAP Assay

The ability of AVE to reduce a ferric-tripyridyltriazine complex to its ferrous form was assessed according to Benzie and Strain [45]. FRAP reagent was prepared by mixing 0.3 mol L^−1^ acetate buffer (pH = 3.6), 10 mmol L^−1^ TPTZ made up in 40 mmol L^−1^ HCl and 20 mmol L^−1^ FeCl_3_ at a 10:1:1 ratio. Then, 0.1 mL of AVE were mixed with 3 mL of the freshly prepared FRAP reagent pre-warmed at 37 °C. The mixture was vortexed, and the absorbance was measured at 593 nm after 30 min of incubation at 37 °C in the darkness. Trolox in EtOH:H_2_O (40%, *v*/*v*) was used as the reference standard (5–100 mg kg^−1^) (R^2^ = 0.9996). The results were expressed as milligrams of trolox equivalents (TE) per gram of AVE. Each extract was analyzed in triplicate.

#### 2.4.4. Aloin Content Determination

The total aloin content (A and B stereoisomers) in AVE was determined as described by Brown et al. [46] with slight modifications. An Agilent series 1260 Infinity Quaternary HPLC system (Agilent Technologies, Palo Alto, CA, USA) equipped with a diode array detector was used. Chromatographic separation was achieved with a Teknokroma Brisa LC2 C_18_ column (150 × 4.6 mm I.D. × 5 µm film thickness). Then, 15 µL of samples were injected, and a gradient of eluent A (water) and eluent B (acetonitrile) was used. The flow rate was 1.0 mL min^−1^ and gradient elution conditions applied were: 17% B for 8 min followed by a linear gradient to 100% B in 20 min (held 1 min), followed by a linear gradient to 17% B in 5 min. Standard solutions were freshly prepared for quantification of aloin A and B (0.005–100 mg kg^−1^, R^2^ = 0.9998). All samples were filtered through 0.45 µm pore size nylon membrane filters prior to HPLC analysis at 357 nm. Triplicate runs were carried out for each sample. Linearity, limit of detection (LOD), limit of quantification (LOQ) and precision were determined to validate the analytical method.

### 2.5. Characterization of AVE Obtained under Optimum Conditions

#### 2.5.1. Attenuated Total Reflectance-Fourier Transform Infrared Spectroscopy (ATR-FTIR)

The FTIR spectrum of AVE was recorded by using an infrared spectrophotometer JASCO FTIR 4700 IRT-5200 (Easton, MD, USA) in the 4000–500 cm^−1^ range with a spectral resolution of 4 cm^−1^ and 32 scans. The attenuated total reflectance (ATR) mode was used with a Golden Gate accessory equipped with diamond crystal, and tests were performed in triplicate.

#### 2.5.2. Thermogravimetric Analysis (TGA)

The TGA of AVE was carried out using Mettler Toledo TGA/SDTA 851e equipment (Schwarzenbach, Switzerland). Approximately 5 mg of the sample were heated from room temperature to 700 °C at 10 °C min^−1^ under nitrogen atmosphere (50 mL min^−1^) using alumina pans. The analysis was performed in triplicate.

#### 2.5.3. AVE Phenolic Profile

##### 2.5.3.1. HPLC-MS Qualitative Analysis

The identification of phenolic compounds present in AVE was performed by high-performance liquid chromatography coupled to mass spectrometry (HPLC-MS) according to Lee et al. [47] and Quispe et al. [48], with some modifications. An Agilent 1100 HPLC system equipped with a quaternary solvent delivery system coupled to an LC/MSD ion trap mass spectrometer with electrospray ionization (ESI) source (Agilent Technologies, Palo Alto, CA, USA) was used. Chromatographic separation was performed on a HALO C_18_ column (100 mm × 4.6 mm I.D. × 2.7 µm) coupled to a HALO C_18_ guard column 90 Å (4.6 × 5 mm I.D. × 2.7 µm) operating at 25 °C. The mobile phase was composed of two solvents added with 0.1% formic acid (A: water, B: acetonitrile). The gradient elution program was as follows: linear gradient from 10% B to 40% B in 20 min followed by a linear gradient to 98% B in 5 min (held 5 min). The flow rate was 0.3 mL min^−1^ and the injection volume 6 µL. MS spectra were recorded in the range 50–900 *m/z* in negative ionization mode. The electrospray chamber was set at 3.5 kV with a drying gas temperature of 350 °C. The N_2_ pressure and flow rate of the nebulizer were 50 psi and 10 L min^−1^, respectively.

AVE and standard solutions of phenolic compounds were freshly prepared in methanol and filtered through a 0.22 µm nylon membrane prior to injection. Extracted ion chromatograms and mass spectra experimental data were used for identification of polyphenols in AVE through comparison with the standards. Other phenolic compounds also present in AVE were tentatively identified based on the information of ion fragments and reported literature.

##### 2.5.3.2. HPLC-DAD Quantitative Analysis

Quantitative analysis of previously identified compounds by HPLC-MS was carried out by HPLC-DAD. An Agilent series 1260 Infinity Quaternary LC HPLC system (Agilent Technologies, Palo Alto, CA, USA) equipped with a diode array detector was used. Operating conditions detailed in Section 2.5.3.1 were applied, and simultaneous monitoring was set at 254 nm (aloesin and aloe-emodin), 270 nm (cinnamic acid), 324 nm (chlorogenic acid) and 357 nm (orientin, aloin B and aloin A) for quantification. The optimal detection wavelength of each compound was set by previously verifying the obtained UV spectra. Quantitative analysis was performed using the external calibration method based on the preparation of calibration curves at ten concentration levels for the aforementioned standard compounds in methanol. All analyses were carried out in triplicate. Linearity, limit of detection (LOD), limit of quantification (LOQ) and precision (RSD) were determined to validate the analytical method.

### 2.6. Scanning Electron Microscopy (SEM)

SEM was employed to evaluate the damage in the vegetal material produced by the MAE process. Raw and extracted AVS freeze-dried samples were mounted on aluminum stubs and then coated with a gold layer under vacuum using an SCD 004 Balzers sputter coater (Bal Tec., AG, Furstentum, Liechtenstein). SEM analysis was carried out using a JEOL JSM 840 scanning electron microscope (Peabody, MA, USA) at an accelerating voltage of 15 kV and 2500× magnification level.

### 2.7. Statistical Analysis

Statgraphics Centurion XVI (Statistical Graphics, Rockville, MD, USA) was used to generate and analyze the BBD results. Graphic analysis of the principal effects and interactions between variables was used to interpret the results. The coefficients of quadratic polynomial models were determined by data regression analysis and the adequacy of the fitted models was determined by evaluating the lack of fit, coefficient of determination (R^2^), and F test obtained from the analysis of variance (ANOVA). The statistical significance of the model parameters was determined at a 95% probability level (α = 0.05).

## 3. Results and Discussion

### 3.1. AVS Characterization

The dimensions of *Aloe vera* leaves used in this work (Table 1) were very similar to the morphological characteristics of plants studied by Zapata et al. [49]. Even though other authors reported different dimensions for three- or four-year old *Aloe vera* plants [3,38,50], it is well known that many factors, such as environmental and soil conditions, water availability and plant variety, could significantly influence plant growth, thereby leading to different leaves in terms of size and phytochemical constituents [51]. In this case, after tissue separation of *Aloe vera* leaves, the yields of skin and other components of the residue, such as the tip, base and spikes, accounted for up to 15.1 ± 2.1% and 17.5 ± 4.1%, respectively. These results are in close agreement with those found by Femenia et al. [38] and Flores-López et al. [3], who reported very similar yields for the epidermis fraction in *Aloe vera* plants. Several drying techniques, including freeze drying, air drying and oven drying at different temperature and time programs, were tested in order to find the most suitable drying process to avoid leaf browning, which might be directly related to oxidative processes and detrimental effects on bioactive substance content in plants. Similar to Ng et al. [52], freeze drying was selected as the most adequate drying process and no apparent oxidation of AVS was observed. On the other hand, it is well known that a smaller particle size will enhance solute–solvent interaction during the extraction process; but too many fine particles might make subsequent separation steps difficult. In this work, particles passing through a 1.0 mm sieve were used.

The physicochemical characterization results of AVS are shown in Table 1. Moisture, ash, protein and fat contents of AVS resulted in close agreement with experimental data reported in other studies [3,12,38]. Proteins and lipids represented a minor fraction of AVS, accounting for 6.5 ± 0.2 wt% and 2.4 ± 0.1 wt%, expressed on a dry matter basis, respectively. On the other hand, the ash content was relatively high, and it accounted for 15.5 ± 0.1%. It has been reported that calcium, potassium, sodium and magnesium are the main mineral components present in AVS ash. It has also been suggested that the presence of minerals in *Aloe vera* is essential for the proper functioning of enzymatic systems and for improving resistance against microorganisms and water stresses. The mineral content found in AVS was much more concentrated compared to other plant tissues, such as gel or flowers [3]. Apart from these components, it has also been reported that AVS is an interesting source of polysaccharides [53] and lignocellulosic matter [12]. Moreover, Lucini et al. [4] and Quispe et al. [48] reported that AVS is rich in phenolic compounds and its extracts exhibit antioxidant activity to a higher degree than other parts of the plant. In this sense, AVS represents a low-cost, underused biomass resource with high potential to be valorized, finding a wide range of applications in cosmetic, food or medicinal products.

### 3.2. MAE Optimization

#### 3.2.1. Model Fitting and Analysis

The optimization of MAE conditions for the extraction of polyphenols from a wide variety of plant leaves, such as *Myrtus communis* L. [54], *Achillea millefolium* [55], strawberry [56], *Vitis vinifera* L. [57] and artichoke [58], has been reported in recent years by using response surface methodology (RSM). Even though these vegetal matrices might have results similar to *Aloe vera* leaf skin, optimized MAE conditions could not be generalized to all plant materials due to the diverse nature of the existing bioactive phytochemicals and the different levels of interaction between microwave irradiation and different plants and vegetable parts. Moreover, it is well known that the MAE of bioactive compounds from plant materials could be affected by a wide number of experimental factors; in consequence, MAE optimization should be performed in each study. In this work, the influence of ethanol concentration (%Et), extraction temperature (T), extraction time (t) and solvent volume (V) on the MAE of bioactive compounds from AVS was studied by using a Box-Behnken experimental design with 29 independent runs, including 5 central points, which were performed randomly. The experimental conditions and data obtained in terms of extraction yield, TPC, antioxidant activity (DPPH and FRAP assays) and total aloin content are shown in Table 2.

Multiple regression analysis was applied to the experimental data, and all the studied responses were fitted to second-order mathematical models as a function of the independent factors. The resulting models are presented in Equations (3)–(7).

(3) $$\begin{array}{ll} \mathrm{Extraction}\;\mathrm{yield}= & -23.762+0.512\;A\;+0.122\;B\;+0.326\;C\;+0.824\;D\;-0.004{\;A}^{2}+0.000\;\mathrm{AB}\;-0.002\;\mathrm{AC}\;\\ & -0.003\;A\;D\;-0.002{\;B}^{2}-0.001\;\mathrm{BC}\;+0.002\;\mathrm{BD}\;-0.001{\;C}^{2}-0.001\;\mathrm{CD}\;-0.005 \end{array}$$

(4) $$\begin{array}{ll} \mathrm{TPC}=98.296 & +0.533\;A\;-0.245\;B\;-0.952\;C\;-0.295\;D\;+0.001{\;A}^{2}+0.001\;\mathrm{AB}\;-0.004\;\mathrm{AC}\;\\ & +0.002\;\mathrm{AD}\;-0.001{\;B}^{2}+0.009\;\mathrm{BC}\;+0.001\;\mathrm{BD}\;-0.006{\;C}^{2}+0.014\;\mathrm{CD}\;-0.002{\;D}^{2} \end{array}$$

(5) $$\begin{array}{ll} \mathrm{DPPH}=52.525 & +0.123\;A\;-0.575\;B\;-0.439\;C\;+0.504\;D\;+0.004{\;A}^{2}-0.002\;\mathrm{AB}\;-0.0025\;\mathrm{AC}\;\\ & -0.003\;\mathrm{AD}\;+0.006{\;B}^{2}+0.011\;\mathrm{BC}\;-0.003\;\mathrm{BD}\;-0.006{\;C}^{2}+0.002\;\mathrm{CD}\;-0.002{\;D}^{2} \end{array}$$

(6) $$\begin{array}{ll} \mathrm{FRAP}=193.053 & -0.317\;A\;-0.490\;B\;-1.384\;C\;-2.129\;D\;-0.001{\;A}^{2}+0.001\;\mathrm{AB}\;+0.014\;\mathrm{AC}\;\\ & +0.012\;\mathrm{AD}\;+0.005{\;B}^{2}+0.009\;\mathrm{BC}\;-0.007\;\mathrm{BD}\;-0.006{\;C}^{2}+0.006\;\mathrm{CD}\;+0.013{\;D}^{2} \end{array}$$

(7) $$\begin{array}{ll} \mathrm{aloin}=56.258 & -0.066\;A\;-0.037\;B\;-0.340\;C\;-0.455\;D\;+0.003{\;A}^{2}-0.003\;\mathrm{AB}\;+0.005\;\mathrm{AC}\;\\ & +0.002\;\mathrm{AD}\;+0.003{\;B}^{2}+0.003\;\mathrm{BC}\;-0.003\;\mathrm{BD}\;-0.004{\;C}^{2}+0.001\;\mathrm{CD}\;+0.004{\;D}^{2} \end{array}$$

where A, B, C and D represent ethanol concentration, extraction temperature, extraction time and solvent volume, respectively.

In order to study the effect of the experimental factors in the response variables as well as to evaluate the adequacy of the fitted models, analysis of variance (ANOVA) was carried out and results are summarized in Table 3. The coefficient of determination (R^2^) values and the *p*-values obtained for lack of fit test were used as a measurement of the degree of fitness of the proposed models to the experimental data. As shown in Table 3, non-significance of the lack of fit test (*p* > 0.05) and acceptable R^2^ values were obtained in all cases, accounting for 0.9498, 0.9308, 0.8691, 0.9329 and 0.9354 for yield, TPC, DPPH, FRAP and aloin content, respectively. These values indicate the percentage of variability being explained by the models for each response (87 to 95%), highlighting a high level of correlation between the experimental data and predicted values. Moreover, adjusted R^2^ values did not differ greatly from R^2^ ones, and in all cases, the distribution of residuals was randomly scattered around zero. These results confirmed the reliability of the models to be used in subsequent optimization stages.

#### 3.2.2. Effect of Extraction Variables on the Extraction Yield

The extraction yield ranged from 18.2 to 26.3 g AVE 100 g AVS^−1^ under the 29 experiments performed (Table 2). According to Table 3, ethanol concentration and solvent volume had significant effects (*p* < 0.05) on extraction yield, with the former being the most significant factor by far. Moreover, three quadratic effects (A^2^, B^2^ and D^2^) also showed significant effects (*p* < 0.05). Particularly, ethanol concentration and all mentioned quadratic effects had negative effects, while solvent volume showed a positive effect. Consequently, significantly higher extraction yields were obtained at a low ethanol concentration level. Solvent constitution is directly related to the type and quantity of compounds extracted from plant materials. In this case, a low ethanol concentration might favor the co-extraction of other untargeted plant components apart from polyphenols. It has been reported that increasing water content in the extraction solvent resulted in higher polarity, which might enhance the extraction of other polar compounds from AVS such as polysaccharides [59]. Furthermore, water addition can be used to improve solvent penetration into vegetal sample matrices as well as to enhance heating efficiency, usually leading to higher extraction yields [26]. On the other hand, extraction yield was significantly affected (*p* < 0.05) by the solvent volume. A positive effect was observed, so the higher the solvent volume, the higher the obtained extraction yield. A low amount of solvent usually promotes mass transfer barriers, which limit the movement of compounds out of the vegetal matrix since active compounds are concentrated in certain regions near the vegetal surface [26]. On the contrary, larger extraction volumes can cause more efficient swelling of plant materials, enhancing the extraction procedure due to an easier rupture of the cell walls. However, in the case of MAE, an excessive amount of solvent may cause poor microwave heating efficiency as the majority of the irradiation would be absorbed by the solvent instead of the plant material and additional power might be needed to obtain higher yields.

#### 3.2.3. Effect of Extraction Variables on Total Phenolic and Aloin Contents

**Figure 1:**
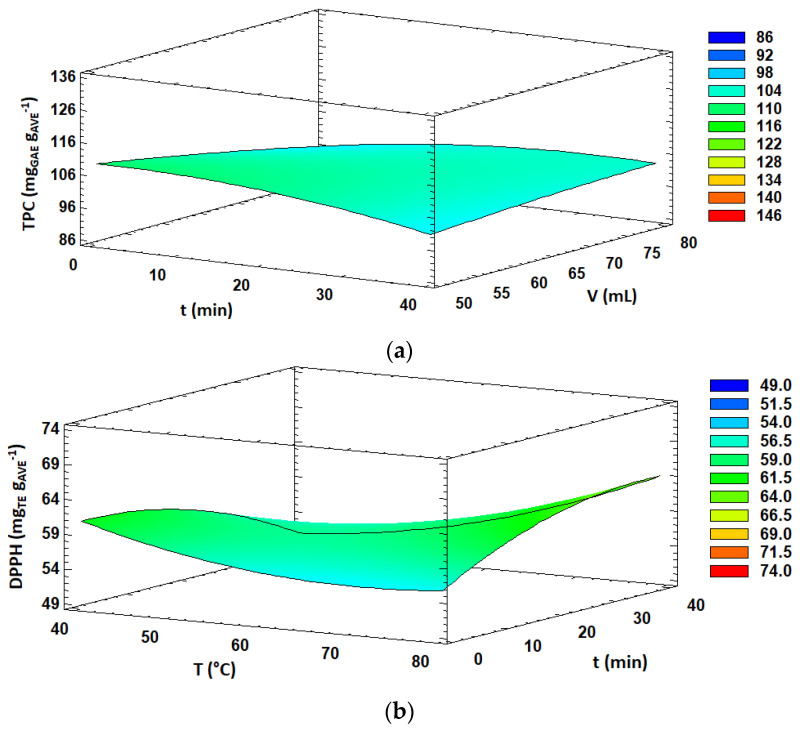

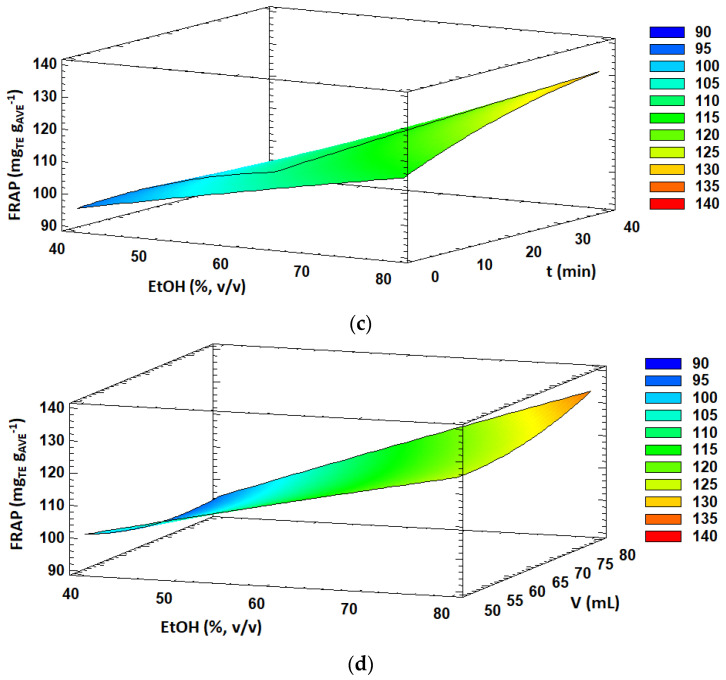
Response surface plots of significant interactions between independent variables on: (**a**) TPC (volume vs. time); (**b**) DPPH (temperature vs. time); (**c**) FRAP (ethanol concentration vs. time) and (**d**) FRAP (ethanol concentration vs. solvent volume). In all cases, the other factors were fixed at their central values.

TPC and aloin content obtained under the 29 experiments carried out in the BBD varied from 86.5 to 125.8 mg GAE g AVE^−1^ and from 36.5 to 57.5 mg g AVE^−1^, respectively (Table 2). ANOVA analysis (Table 3) showed that total phenolic content was significantly (*p* < 0.05) influenced by the ethanol concentration and the interaction between extraction time and solvent volume, both with positive effects, while the aloin content was only significantly affected (*p* < 0.05) by the ethanol concentration, also with a positive effect. Several authors reported that high ethanol concentrations in the extraction solvent were adequate for obtaining extracts with high TPC or antioxidant activities from aloe [4,13,43,44,60] and other plant materials [40,55,61]. The solubility of phenolic compounds is strongly related to their chemical nature and polarity. By increasing ethanol content in the extraction solvent, the solubility of certain polyphenols resulted in enhanced and, consequently, higher TPC and antioxidant activities, as expected. The significant interaction effect (*p* < 0.05) between extraction time and solvent volume on the TPC response is shown in Figure 1a. It was observed that the combination of short extraction times and low solvent volumes resulted in higher TPC values. Similar results were found by other authors who reported that the increase in the exposure time of the sample to microwave irradiation might promote the extraction of other components, apart from polyphenols, from plant materials, causing a relative decrease in TPC values on extracts [55,62]. Furthermore, microwave irradiation time may lead to thermal degradation and oxidation of sensitive compounds such as some polyphenols.

#### 3.2.4. Effect of Extraction Variables on Antioxidant Activity

The antioxidant activity, expressed as DPPH radical scavenging activity, varied from 49.9 to 73.4 mg TE g AVE^−1^ in the 29 MAE experiments carried out in this study (Table 2). DPPH radical scavenging activity was significantly affected (*p* < 0.05) by three linear effects (ethanol concentration, extraction temperature and solvent volume), three quadratic effects (B^2^, C^2^ and A^2^) and one interaction effect (BC). The linear effects of the ethanol concentration and extraction temperature were positive, while the solvent volume showed a negative effect. Furthermore, all quadratic effects were positive except for extraction time. Overall, the DPPH radical scavenging activity increased with increasing ethanol concentration and extraction temperature, while decreasing solvent volume. Moreover, the interaction effect between the extraction temperature and time showed a significant (*p* < 0.05) positive effect on DPPH antioxidant activity. As shown in Figure 1b, an increase in extraction time at low extraction temperature values led to a decrease in DPPH antioxidant activity of the extract, whereas increasing extraction time at high extraction temperatures led to enhanced DPPH values. Overall, higher antioxidant activity was obtained at 80 °C and 40 min. It is well known that polyphenols could exert antioxidant activity and many other interesting properties [63]. In this case, similarly to TPC, a high ethanol concentration favored the extraction of certain components which might act as proton-donor substrates, scavenging the radicals which are involved in DPPH reactions and contributing to the antioxidant activity of AVE. On the other hand, high extraction temperatures caused a drop in viscosity and surface tension, enhancing the capacity of the extraction solvent to penetrate the vegetal matrix, resulting in an increase in the solubility of the target compounds [26]. In addition, cell walls might be damaged by the action of high temperatures during the extraction process, which could favor the release of active compounds in the extraction solvent [26,28]. However, it has been reported that some phenolic compounds might suffer thermal degradation when exposed to high temperatures [64], and optimal temperatures for MAE usually do not exceed 100 °C.

Concerning the antioxidant activity estimated by the FRAP method, the obtained values ranged from 90.5 to 134.1 mg TE g AVE^−1^ under the 29 experiments performed in the BBD (Table 2). FRAP values were significantly influenced (*p* < 0.05) by the linear effect of the solvent concentration and extraction time, quadratic effect of solvent volume and interaction effects of AC and AD. Overall, the significant effects (*p* < 0.05) were positive, resulting in enhanced FRAP antioxidant activity values by increasing ethanol concentration in the extraction solvent, extraction time and solvent volume. Moreover, the response surface plots of the interaction effects between the ethanol concentration and extraction time and solvent volume are shown in Figure 1c,d, respectively. A slight decrease in FRAP values was observed with increasing extraction time at low ethanol concentrations in the extraction solvent. However, FRAP antioxidant activity was enhanced with increasing extraction time at high ethanol concentrations. Similar behavior was observed for the interaction effect between the ethanol concentration and solvent volume. Altogether, higher FRAP values were obtained at high ethanol concentration levels and long extraction times as well as high solvent volumes.

As already detailed for TPC and DPPH values, a high ethanol concentration also favors the extraction of some components, which contributes to enhancing FRAP antioxidant activity. Similar to the extraction temperature, higher extraction times contribute to cell wall degradation and liberation of active compounds into the extraction solvent, increasing the antioxidant activity of the extracts. On this point, it is worth mentioning that different methods used to determine the antioxidant activity of AVE might evaluate different fractions of antioxidant compounds, which might be partially overlapped. On the one hand, the FRAP method evaluates the content of electron-donating species with a certain redox potential, whereas the DPPH method assesses the free radical scavenging capacity of a sample [43]. Moreover, the steric accessibility of DPPH radicals is a major aspect of the reaction, as this method is much more sensitive to small molecules, and many large antioxidant compounds might hardly participate in this reaction [65]. This effect was already highlighted by Kim et al. [43], who extracted polyphenols and antioxidant compounds from *Aloe vera* gel, and they observed that the antioxidant activity of aloe extracts evaluated by the DPPH method may be underestimated to a certain degree.

#### 3.2.5. Optimal Extraction Conditions

Table 4 shows the experimental conditions which optimized each response variable individually and the optimum predicted values by the studied models. Great variability was found among the different sets of experimental factors which maximized each response variable, as expected from the analysis of linear, quadratic and interaction effects of the extraction variables. Consequently, it was not possible to clearly determine the best extraction conditions for AVS only considering these results. For this reason, a simultaneous optimization procedure using the desirability function was carried out to find the experimental conditions which simultaneously satisfy all the requirements for each response. First, the optimization was performed in order to simultaneously maximize extraction yield, TPC, DPPH and FRAP and minimize aloin content. However, due to the dissimilar extraction conditions summarized in Table 4, a quite low desirability value was obtained (0.5802) and further approaches were considered. According to ANOVA analysis (Table 3), the ethanol concentration was by far the most significant (*p* < 0.05) factor affecting all responses. Moreover, it exhibited a strong negative effect on the extraction yield with the optimum at 40%, whereas its effect was positive in all the other responses with an optimum at 80%. These results indicated an inverse correlation between extraction yield and the rest of the responses, suggesting that MAE conditions leading to high extraction yields could not be selective for polyphenol extraction. A similar scenario was described by Kim et al. [43] and Milutinovic et al. [66], who optimized the MAE of antioxidants from *Aloe vera* gel and *Equisetum arvense* waste, respectively. In this sense, considering that extraction yield could be interfered with by the co-extraction of other untargeted compounds and that TPC and antioxidant activity responses are usually directly correlated with phenolic compound concentrations, the extraction yield from the multi-response optimization was excluded.

In addition, it has been reported that aloin contributes to some extent to the antioxidant capacity exhibited by extracts from different aloe species [4,47,67]. Consequently, maximization of antioxidant activity and total phenolic content while simultaneously minimizing aloin content might lead to unavoidably low desirability values (0.5951). Therefore, multiresponse optimization was finally performed, maximizing TPC, DPPH, FRAP and aloin content and obtaining a desirability value of 0.8777, with optimal extraction conditions of 80% ethanol, 80 °C, 36.6 min and 50.0 mL. Subsequent purification or selective extraction steps could be implemented in the case of limitations regarding aloin content.

#### 3.2.6. Verification Test under Optimum Extraction Conditions

The predicted values determined at a 95% level of probability for response variables obtained after multiresponse optimization in terms of extraction yield, TPC, DPPH, FRAP and aloin content were 16.0 ± 2.2 g AVE 100 g AVS^−1^, 118.4 ± 11.9 mg GAE g AVE^−1^, 74.2 ± 8.4 mg TE g AVE^−1^, 134.8 ± 12.0 mg TE g AVE^−1^ and 56.6 ± 5.6 mg aloin g AVE^−1^, respectively. Verification experiments under the obtained optimal conditions were carried out, in triplicate, and the obtained experimental responses regarding extraction yield, TPC, DPPH, FRAP and aloin were 17.3 ± 0.1 g AVE 100 g AVS^−1^, 116.4 ± 4.5 mg GAE g AVE^−1^, 69.0 ± 1.9 mg TE g AVE^−^^1^, 131.9 ± 6.5 mg TE g AVE^−1^ and 55.6 ± 0.2 mg aloin g AVE^−1^, respectively. Experimental results not significantly differing (*p* > 0.05) from predicted values were obtained in all cases. Moreover, reproducibility of the whole AVE extraction and characterization process was demonstrated, obtaining relative standard deviations ranging from 0.36 to 4.92% for all analyzed response variables. Additionally, it was observed that TPC, FRAP and aloin content in optimized AVE were almost higher than every individual run of the design, while DPPH was surpassed by only one experiment (Table 2). Regarding extraction yield, a value near 17% was obtained, as expected, due to the high ethanol concentration used in the extraction solvent. In conclusion, the obtained quadratic models were reliable for optimizing the extraction of bioactive compounds from *Aloe vera* skin in the studied experimental domain, resulting in a high degree of correlation between the experimental data and predicted values, indicating that the developed models could be used to predict the studied responses.

Compared to conventional extraction techniques, MAE’s major advantages are its high reproducibility and the noticeable reduction in extraction time and solvent consumption [26]. For instance, Sultana et al. [60] studied the effect of the extraction solvent on the yield of antioxidant compounds from different plant materials using conventional extraction techniques. They found that reflux extraction of *Aloe barbadensis* leaves with 200 mL of 80% ethanol accounted for 18.1 ± 0.7 g 100 g DW. However, a much longer extraction time of 6 h was required. In another work, Quispe et al. [48] reported an extraction yield of 16.2 g 100 g of AVS for a phenolic-enriched extract obtained after maceration with methanol for 48 h.

### 3.3. Characterization of AVE Obtained at Optimal Extraction Conditions

#### 3.3.1. FTIR Analysis

**Figure 2:**
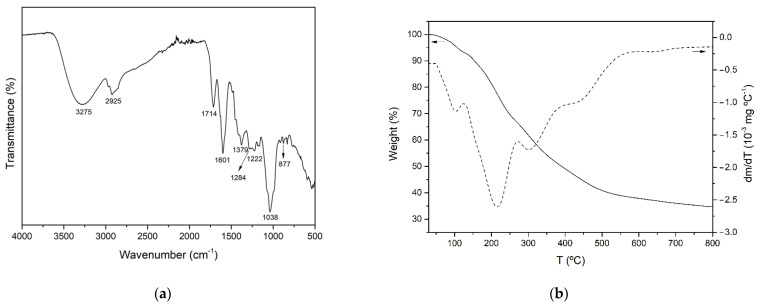
FTIR spectrum (**a**) and TGA and DTGA thermograms (**b**) of AVE obtained under optimal MAE conditions.

It has been reported that *Aloe vera* plants contain a wide variety of bioactive compounds, including phenolic acids and derivatives, flavonoids, chromones, anthraquinones, polysaccharides and fatty acids. The FTIR–ATR spectrum of AVE obtained under optimal extraction conditions is shown in Figure 2a. The broad peak observed around 3275 cm^−1^ was attributed to the stretching vibration of different –OH groups of phenolic compounds such as flavonoids, anthraquinones and chromones [68,69]. The bands at 2925 and 1379 cm^−1^ might be assigned to C-H stretching and bending vibration of aliphatic hydrocarbons, respectively [68,70,71]. The absorption band at 1714 cm^−1^ was associated with C=O stretching, indicating the presence of carbonyl functional groups [70,72]. Peaks observed at 1222 and 877 cm^−1^ might be related to the stretching of C-O-C bonds of acetyl groups of esters and phenols [68,72] and out of plane deformation of C-H bonds in aromatic rings [72], respectively. In addition, according to other authors who characterized polyphenol-enriched extracts from other plant materials, such as *Ilex paraguarensis* [71], *Rosmarinus officinalis* [73], *Garcinia mangostana* [74] and grape seeds [75], the observed peaks at 1601, 1284 and 1036 cm^−1^ could be attributed to C=C ring stretching vibration [71,73,75], ester C-O stretching [75] or the presence of methoxy groups [74], and C-N stretching vibration of aliphatic amines [71], respectively. As a result of the aforementioned peak assignations, it was concluded that AVE contained a variety of phenolic compounds.

#### 3.3.2. Thermogravimetric Analysis (TGA)

The thermal stability of AVE obtained under optimized MAE conditions was studied by thermogravimetric analysis. Figure 2b shows the TGA and DTGA of AVE obtained under an inert nitrogen atmosphere. The thermal degradation process occurred over a wide range of temperatures, showing multiple overlapped decomposition steps which are in accordance with the complex composition of AVE. Similar behavior was reported for the thermal degradation process of other natural extracts, such as those from cocoa shell bean [5], grape seed [75] and yerba mate [71]. According to Figure 2b, the initial step of weight loss started below 100 °C, and it could be associated with water and volatile phenolic compound desorption. The subsequent degradation stages with peaks shown at 215.0 ± 0.4, 301.6 ± 2.0 and 442.1 ± 1.3 °C were relatively overlapped, and they were attributed to the thermal degradation of groups of biomolecules with different structural features and characteristics present in AVE. The second degradation step occurred from 125 to 272 °C with an associated mass loss of 27.3%, and it could be related to the degradation of some low to mid molecular weight components of AVE, such as the compounds which will be further discussed in Section 3.3.3. The third and fourth thermal degradation stages extended from 272 to 400 °C and from 400 to 800 °C with mass losses of 17.3 and 14.7%, respectively. These degradation processes may be ascribed to compounds with higher molecular weights and more complex structures such as cellulose and lignin derivatives that might be present in *Aloe vera* extracts [70]. Finally, nearly 34.1 ± 0.1% of the initial weight remained at 800 °C as char residue, which could be related to non-pyrolyzable compounds.

#### 3.3.3. Determination of Phenolic Profile by HPLC

**Figure 3:**
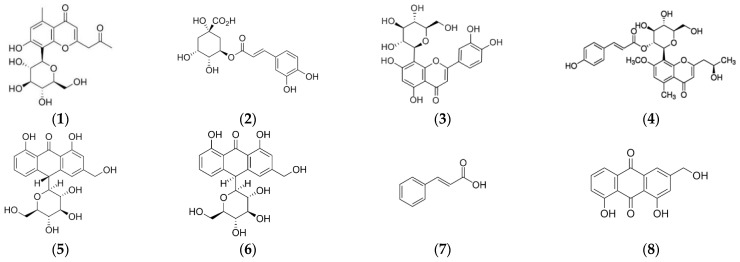
Chemical structures of eight identified compounds by HPLC-MS in AVE. (**1**) aloesin; (**2**) chlorogenic acid; (**3**) orientin; (**4**) aloeresin D; (**5**) aloin B; (**6**) aloin A; (**7**) cinnamic acid; (**8**) aloe emodin.

Optimization of chromatographic conditions (flow rate, mobile phase composition and gradient step) was performed on AVE in order to obtain an adequate peak separation and resolution. Contrary to some authors who reported that a methanol–water mobile phase added with formic or acetic acid was suitable for phenolic profiling of different aloe species [76,77,78], in this study it was found that peak shape and separation efficiency of phenolic compounds in AVE were significantly improved by using an acetonitrile–water mixture, which was selected as the mobile phase in accordance with previous works [47,48,79]. Aloesin, chlorogenic acid, orientin, aloeresin D, aloin B, aloin A, cinnamic acid and aloe-emodin, whose chemical structures are shown in Figure 3, were identified in AVE obtained under optimal MAE conditions by HPLC-ESI-MS/MS by comparing their retention times and mass spectra with those of commercial standards. These results are in general agreement with previous works reporting the presence of these compounds in *Aloe vera* extracts [47,48,76]. It has been stated that the beneficial health-promoting properties found in Aloe leaves might be related to the highly complex phytochemical composition and, in particular, to characteristic phenolic compounds which could act against free radical and oxidative processes [80]. However, a general consensus on the role of some of these phenolic compounds has not been achieved yet, and some studies have highlighted their potential adverse effects [20].

Even though some authors have reported that Aloe contains many common phenolic acid derivatives (gallic acid, protocatechuic acid, syringic acid, gentisic acid), hydroxycinnamic acid derivatives (p-coumaric acid, ferulic acid, caffeic acid, sinapic acid) and flavonoids (naringenin, catechin, epicatechin, kaempferol, rutin, luteolin, myricetin) [4,42,81], only a fraction of the targeted standards was identified in AVE. This fact could be related to the influence of some environmental conditions, such as water and sunlight availability, soil characteristics (pH, conductivity, total N), geographical origin, plant variety and age, harvest season, position of the leaves in the plant and post-harvest treatment, among many other factors, which combined with the effect of the extraction technique could significantly affect secondary metabolite composition in plants [51,79,82]. In addition, it was noticed that *m*/*z* signals corresponding to several aforementioned compounds were recorded at different retention times compared to the standards, suggesting that isomeric compounds of target polyphenols might be present in AVE.

**Figure 4:**
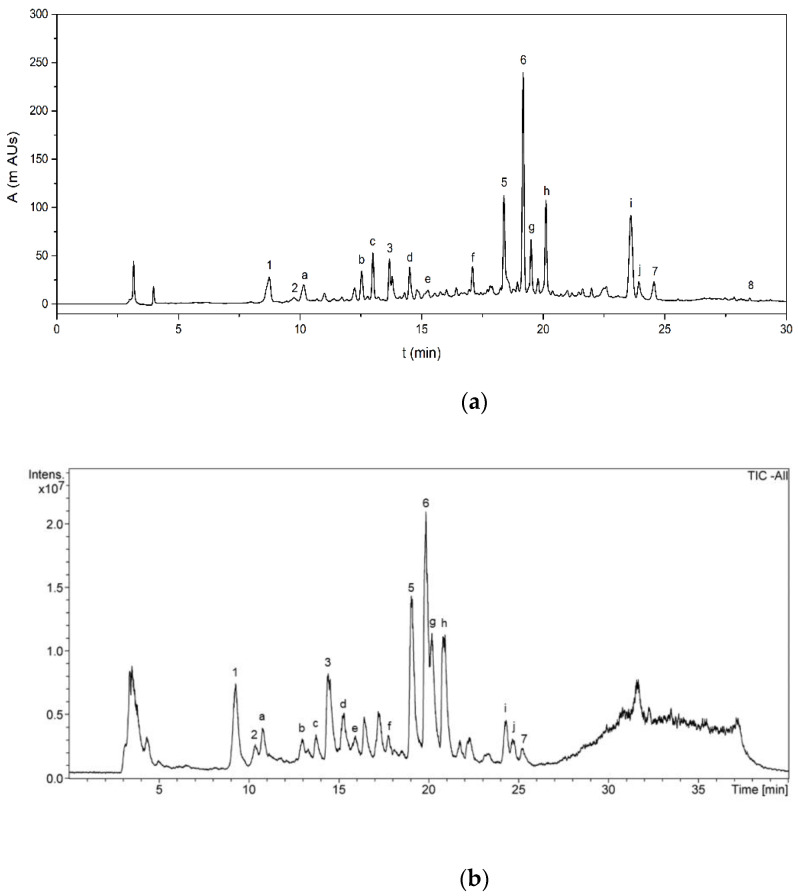
(**a**) HPLC-DAD chromatogram showing the phenolic profile of AVE obtained under optimum MAE conditions; (**b**) HPLC-MS total ion chromatogram (TIC) of AVE. See Table 5; Table 6 for peak assignations.

The phenolic compounds identified in AVE were quantified by HPLC-DAD (Figure 4a). Calibration curves of aloesin, chlorogenic acid, orientin, aloeresin D, aloin B, aloin A, cinnamic acid and aloe-emodin were obtained, showing acceptable levels of linearity with determination coefficients (R^2^) ranging from 0.9960 to 0.9999 for all the analyzed standards at ten concentration levels (Table 5). The LOD and LOQ values that were obtained ranged from 0.018 to 0.383 mg kg^−1^ and from 0.061 to 1.275 mg kg^−1^, respectively, indicating the feasibility of the proposed method for the quantification of the identified polyphenols. Finally, precision in terms of repeatability was evaluated by analyzing standard solutions, in triplicate, for all concentration levels within the same day, with relative standard deviations ranging from 0.9 to 2.6%, showing good repeatability. Diastereomeric anthraquinone derivatives Aloin A and B were the main components present in AVE, accounting for 702 ± 2 and 308.1 ± 0.6 mg 100 g AVS^−1^, respectively. Another major component found in AVE was the chromone aloesin with a concentration of 292.6 ± 0.5 mg 100 g AVS^−1^. Other quantified phenolic compounds ranged from 3.6 ± 0.1 to 80.0 ± 0.2 mg 100 g AVS^−1^. Quantification results and analytical figures of merit for HPLC-DAD analysis are summarized in Table 5.

Phenolic compounds present in *Aloe vera* plant have been extensively studied in recent decades due to their well-known beneficial and health promoting properties. However, the vast majority of the research was focused on the inner gel of the plant and very few reports on *Aloe vera* skin phenolic profiles and contents are currently available. Añibarro-Ortega et al. [13] reported that chromones and anthrones accounted for up to 44.9% and 43.8% of the phenolic compounds found in *Aloe vera* rind extract, respectively, with the concentration of aloesin, aloin B and aloin A being 34.4 ± 0.7, 4.3 ± 0.3 and 9.9 ± 0.4 mg g AVE^−1^, respectively. The relative concentration reported for aloin A and B diastereomers is in close agreement with the results shown in Table 5, and aloin A was 2.3 times more concentrated than aloin B, although smaller quantities of both aloins were reported. On the other hand, López et al. [42] found a lower chlorogenic acid concentration in AVS, accounting for only 7.8 ± 0.2 mg 100 g AVS^−1^. Lucini et al. [4] suggested that hydroxycinnamic acids, anthrones and chromones might have a direct and relevant role in the antioxidant potential of *Aloe vera* extracts.

Other studies reported quantitative contents of aloesin, aloeresin D, aloin A, aloin B, aloe-emodin, orientin, cinnamic acid and chlorogenic acid [76,78,83,84,85,86]. However, different plant species, Aloe plant tissues (i.e., whole leaf, gel, exudate) and processing and extraction techniques were used, and comparison of quantification results might not be straightforward. For instance, Lai et al. [83] reported 4.26 ± 0.14 and 25.81 ± 0.21 mg 100 g FW^−1^ of whole *Aloe barbadensis* leaf for chlorogenic acid and total aloin, respectively. Moreover, Cardarelli et al. [86] analyzed exudates from six-year-old *Aloe barbadendis* plants and found 2.81± 0.21, 0.042 ± 0.007, 0.016 ± 0.001, 1.96 ± 0.40 and 3.27 ± 0.20 100^−1^ g of aloin, aloe-emodin, aloenin, aloesin and aloeresin A, respectively. Similarly, Wu et al. [76] found that the main component present in *Aloe barbadensis* exudate was aloin A, accounting for 178 to 219 mg g^−1^. Moreover, aloesin and aloe-emodin contents ranged from 0.90 to 19.3 and from 0.96 to 2.27 mg g^−1^, respectively. On the other hand, Aldayel et al. [84] reported 43.3 ± 2.8 and 21.4 ± 2.0 mg g^−1^ of *Aloe vera* gel extract for aloin B and A, respectively. In conclusion, a wide range of quantitative results could be found in the literature using different approaches for the analysis of *Aloe vera* plant components.

Apart from the eight already quantified phenolic compounds, other peaks were also detected in the negative ion mode as [M-H],^-^ and they were tentatively identified based on their accurate mass fragments, elution sequence and careful analysis of the literature (Table 6). A typical total ion chromatogram (TIC) of AVE obtained by HPLC-MS is shown in Figure 4b. The MS spectrum found for peak a showed the most abundant fragment at *m*/*z* 455. Añibarro-Ortega et al. [13] reported similar findings, although the structure of this compound has not yet been elucidated. Peak b eluted at 13.0 min, showing fragments at *m*/*z* 337 and 609, which were tentatively identified as cis or trans-5-p-coumaroylquinic acid and luteolin-6,8-C-diglucoside, respectively, by the same authors. The mass spectrum for peak c exhibited a main fragment at *m*/*z* 447, revealing a group of orientin isomers which, according to the literature, might be identified as 8-*O*-methyl-7-hydroxyaloin [47,84] or luteolin-6-C-glucoside [13]. Peaks d and e showed a main fragment at *m*/*z* 433. It has been reported that *Aloe vera* extracts contain aloin-derived diastereoisomers such as 5, 7 or 10-hydroxyaloin, which were proposed for the tentative identification of these peaks [13,47,48,76,85]. Similarly, peaks f, g, h and j were tentatively identified as dihydroisocoumarin glucoside [84], 6′-malonylnataloin B [47,48], 6′-malonylnataloin A [47,48] and 5,3′-dihydroxy-6,7,4′-trimethoxyflavone [48], respectively. Finally, peak i showed a main fragment at *m*/*z* 585. A compound with 585 g mol^−1^ of molecular weight was also found by Añibarro-Ortega et al. [13], although its molecular structure was not determined.

**Table 6:** Tentative identification of unknown phenolic compounds in AVE by HPLC-MS.

Peak ^1^	t_R_ ^2^	(*m*/*z*)	Elemental Composition	Tentative Identification	Ref.
	(min)	[M-H]^-^			
a	10.9	455	-	Unknown	[13]
b	13	337	C_16_H_17_O_8_^-^	cis or trans 5-p-Coumaroylquinic acid	[13]
		609	C_27_H_29_O_16_^-^	luteolin-6,8-*C*-diglucoside	[13]
c	13.8	447	C_22_H_23_O_10_^-^	8-*O*-methyl-7-hydroxyaloin	[47,84]
		447	C_22_H_23_O_10_^-^	luteolin-6-*C*-glucoside	[13]
d	15.2	433	C_21_H_21_O_10_^-^	7-hydroxyaloin B	[47]
		433	C_21_H_21_O_10_^-^	10-hydroxyaloin B	[13,48,76,85]
		433	C_21_H_21_O_10_^-^	5-hydroxyaloin B	[13]
e	15.9	433	C_21_H_21_O_10_^-^	7-hydroxyaloin A	[47]
		433	C_21_H_21_O_10_^-^	10-hydroxyaloin A	[13,48,76,85]
		433	C_21_H_21_O_10_^-^	5-hydroxyaloin A	[13]
f	17.7	505	C_24_H_25_O_12_^-^	Dihydroisocoumarin glucoside	[84]
g	20.2	459	C_23_H_23_O_10_^-^	6′-malonylnataloin B	[47,48]
h	20.8	459	C_23_H_23_O_10_^-^	6′-malonylnataloin A	[47,48]
i	24.2	585	-	Unknown	[13]
j	24.7	343	C_18_H_15_O_7_^-^	5,3′-Dihydroxy-6,7,4′-trimethoxyflavone	[48]

^1^ Notation for peak identification refers to Figure 4. ^2^ Retention times from HPLC-MS according to extracted ion chromatograms (EICs).

### 3.4. Scanning Electron Microscopy (SEM)

**Figure 5:**
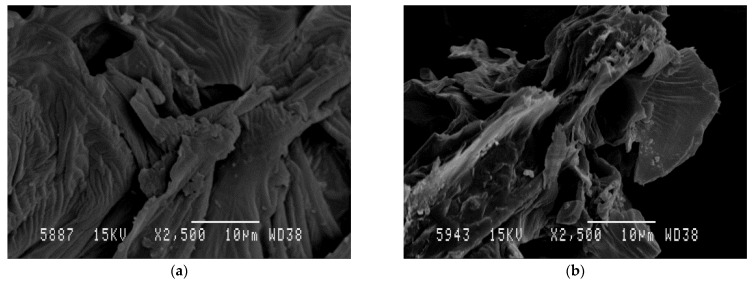
SEM micrographs of raw AVS (**a**) and AVS after MAE under optimal conditions (**b**).

SEM was used to investigate the impact of the MAE process on the AVS structure. Figure 5a,b show SEM micrographs obtained for raw AVS after drying and grinding steps as well as for AVS residue obtained after MAE under optimal conditions, respectively. A significant change in the surface morphology of the AVS particles was observed. Before extraction, particles exhibited a relatively smooth and unaltered surface. However, after MAE, AVS showed increased porosity and evidenced surface deterioration. These results are in accordance with other authors reporting similar effects of microwave irradiation on diverse vegetal materials such as cocoa bean shell [5], *Pinus radiata* bark [87] and brown seaweeds [88]. In all cases, it was suggested that the damage to the vegetal structure can facilitate solvent penetration, enhancing diffusion and extraction of bioactive compounds, which helps to increase TPC and antioxidant activity values.

## 4. Conclusions

A new MAE methodology was developed for the extraction of bioactive compounds from *Aloe vera* skin wastes as a green and fast strategy for the valorization of these agrowastes. The combined effects of MAE experimental parameters such as ethanol composition, temperature, time and solvent volume on extraction yield, TPC, DPPH, FRAP and aloin content of the extracts were studied and optimized by using a BBD. Second-order polynomial regression models with high reliability were obtained and MAE conditions which simultaneously optimize all responses were 80% ethanol, 80 °C, 36.6 min and 50.0 mL. Under these extraction conditions, the obtained responses regarding extraction yield, TPC, DPPH, FRAP and aloin were 17.3 ± 0.1 g AVE 100 g AVS^−1^, 116.4 ± 4.5 mg GAE g AVE^−1^, 69.0 ± 1.9 mg TE g AVE^−1^, 131.9 ± 6.5 mg TE g AVE^−1^ and 55.6 ± 0.2 mg aloin g AVE^−1^, respectively. Structural (FTIR) and thermal (TGA) characterization results were in accordance with AVE composition, while significant differences in surface morphology were evidenced by SEM in AVS before and after MAE. Moreover, eight major phenolic compounds (aloesin, chlorogenic acid, orientin, aloeresin D, aloin B, aloin A, cinnamic acid and aloe-emodin) were identified and quantified by HPLC-DAD/MS, while eight other compounds were also tentatively identified. Diastereomeric anthraquinone derivatives Aloin A and B were the main components present in AVE, followed by the chromone aloesin. According to the obtained results, the proposed method could be a promising procedure for obtaining antioxidant extracts rich in polyphenols with potential industrial applications in the food, biomedical or cosmeceutical industries, as well as contributing to the circular economy and reducing food waste and environmental impact issues.

## Figures and Tables

**Table 1:** Leaf dimensions, *Aloe vera* waste yield and chemical characterization of *Aloe vera* skin (AVS). Results are expressed as mean ± SD.

**Leaf Dimensions ^1^**	
Length (cm)	64.9 ± 3.7
Width at base (cm)	12.8 ± 0.8
Thickness (cm)	2.8 ± 0.3
**Waste yield ^1,2^**	
Weight (g)	780 ± 90
Skin waste (%)	15.1 ± 2.1
**Chemical characterization of AVS ^3^**	
Moisture (g 100 g FW^−1^)	84.9 ± 0.8
Ash (g 100 g DW^−1^)	15.5 ± 0.1
Protein (g 100 g DW^−1^)	6.5 ± 0.2
Lipids (g 100 g DW^−1^)	2.4 ± 0.1

^1^*n* = 100; ^2^ Expressed as a fraction of the total leaf weight; ^3^ *n* = 3. FW: fresh weight; DW: dry weight.

**Table 2:** Box-Behnken experimental design matrix and response values obtained from *Aloe vera* skin extracts using microwave-assisted extraction (MAE).

	Experimental Domain				Response Variables				
Run	Et (%, *v*/*v*)	T (°C)	t (min)	V (mL)	Yield (gAVE 100 gAVS^−1^)	TPC (mgGAE gAVE^−1^)	DPPH (mgTE gAVE^−1^)	FRAP (mgTE gAVE^−1^)	Aloin (mg gAVE^−1^)
1	60	80	22.5	80	24.2	104.9 ± 1.8	59.6 ± 5.1	110.7 ± 3.2	46.1 ± 0.2
2	40	60	5.0	65	24.3	86.5 ± 0.9	51.4 ± 6.1	95.2 ± 3.7	39.2 ± 0.2
3	60	80	22.5	50	18.8	102.8 ± 1.4	65.0 ± 6.3	120.2 ± 4.2	50.3 ± 0.1
4	80	60	22.5	50	18.4	122.4 ± 1.2	68.4 ± 5.3	119.3 ± 3.2	53.0 ± 0.3
5	40	60	22.5	50	22.9	91.1 ± 0.4	53.5 ± 6.2	101.7 ± 1.7	42.0 ± 0.1
6	60	60	40.0	50	22.5	102.4 ± 0.5	58.7 ± 4.9	109.9 ± 3.8	42.7 ± 0.2
7	40	80	22.5	65	25.4	91.0 ± 0.9	61.6 ± 5.2	93.0 ± 2.0	39.0 ± 0.2
8	60	40	22.5	50	21.1	101.3 ± 0.5	54.9 ± 5.9	116.2 ± 3.5	48.2 ± 0.3
9	80	60	22.5	80	18.2	121.3 ± 1.7	61.6 ± 4.5	132.1 ± 0.7	54.2 ± 0.3
10	80	60	40.0	65	18.7	114.9 ± 1.6	59.3 ± 5.0	130.7 ± 3.6	56.6 ± 0.5
11	60	60	22.5	65	24.2	105.4 ± 1.1	58.3 ± 4.2	110.5 ± 5.7	46.9 ± 0.4
12	60	40	40.0	65	23.2	103.6 ± 1.0	54.4 ± 6.5	108.7 ± 4.2	44.5 ± 0.3
13	60	60	22.5	65	23.3	102.2 ± 1.1	57.8 ± 2.1	113.8 ± 3.0	46.1 ± 0.0
14	60	60	5.0	80	23.9	98.5 ± 1.1	55.2 ± 5.5	113.6 ± 3.9	45.4 ± 0.2
15	80	40	22.5	65	18.8	121.4 ± 1.1	68.1 ± 5.1	134.1 ± 2.8	57.5 ± 0.2
16	60	60	22.5	65	23.6	104.0 ± 1.3	55.2 ± 4.9	113.3 ± 4.3	47.1 ± 0.4
17	80	60	5.0	65	20.3	117.9 ± 1.2	64.3 ± 4.3	116.3 ± 5.1	52.9 ± 0.1
18	60	80	40.0	65	22.7	103.6 ± 1.4	62.5 ± 4.4	118.8 ± 1.9	47.1 ± 0.1
19	80	80	22.5	65	19.4	125.8 ± 1.3	73.4 ± 4.6	131.5 ± 6.0	53.3 ± 0.3
20	40	60	40.0	65	25.1	89.3 ± 0.4	49.9 ± 5.4	90.5 ± 2.4	36.5 ± 0.1
21	40	60	22.5	80	26.3	87.4 ± 0.7	50.5 ± 5.6	99.6 ± 2.5	41.0 ± 0.1
22	60	60	5.0	50	21.0	116.3 ± 2.2	62.1 ± 2.9	110.4 ± 1.8	44.8 ± 0.4
23	60	60	22.5	65	24.7	106.4 ± 0.9	58.8 ± 6.1	108.2 ± 1.0	43.5 ± 0.2
24	60	40	22.5	80	23.4	102.7 ± 2.4	53.5 ± 6.2	114.6 ± 5.4	47.6 ± 0.5
25	60	60	22.5	65	24.9	108.7 ± 2.3	59.1 ± 4.7	109.6 ± 9.5	43.0 ± 0.3
26	60	40	5.0	65	22.1	109.4 ± 1.2	61.2 ± 7.0	109.3 ± 0.2	45.6 ± 0.3
27	40	40	22.5	65	24.8	88.8 ± 0.3	53.9 ± 6.2	97.7 ± 3.8	38.4 ± 0.3
28	60	60	40.0	80	23.8	99.1 ± 0.6	54.1 ± 4.2	119.7 ± 14.1	44.3 ± 0.1
29	60	80	5.0	65	22.8	96.8 ± 1.2	53.4 ± 5.8	106.3 ± 3.5	44.4 ± 0.4

Et: ethanol concentration; T: extraction temperature; t: extraction time; V: solvent volume. AVS: *Aloe vera* skin; AVE: *Aloe vera* extract; GAE: gallic acid equivalents; TE: trolox equivalents.

**Table 3:** ANOVA results for response surface quadratic models of AVS extraction.

Source	Sum of Squares	Df	Mean Square	F-Value	*p*-Value
**Yield**					
A	102.08	1	102.08	215.82	0.0001 *
B	0.00	1	0.00	0.00	0.9685
C	0.21	1	0.21	0.45	0.5386
D	19.00	1	19.00	40.17	0.0032 *
AA	13.00	1	13.00	27.49	0.0063 *
AB	0.00	1	0.00	0.00	1.0000
AC	1.44	1	1.44	3.04	0.1560
AD	3.24	1	3.24	6.85	0.0590
BB	5.29	1	5.29	11.19	0.0287 *
BC	0.36	1	0.36	0.76	0.4322
BD	2.40	1	2.40	5.08	0.0873
CC	1.26	1	1.26	2.67	0.1779
CD	0.64	1	0.64	1.35	0.3094
DD	9.01	1	9.01	19.04	0.0120 *
Lack-of-fit	6.02	10	0.60	1.27	0.4399
Pure error	1.89	4	0.47		
Total (corr.)	157.55	28			
R^2^	0.9498				
Adj R^2^	0.8995				
**TPC**					
A	2995.68	1	2995.68	497.79	0.0000 *
B	0.44	1	0.44	0.07	0.8001
C	13.02	1	13.02	2.16	0.2153
D	41.81	1	41.81	6.95	0.0578
AA	1.15	1	1.15	0.19	0.6842
AB	1.21	1	1.21	0.20	0.6771
AC	8.41	1	8.41	1.40	0.3026
AD	1.69	1	1.69	0.28	0.6242
BB	0.55	1	0.55	0.09	0.7777
BC	39.69	1	39.69	6.60	0.0621
BD	0.12	1	0.12	0.02	0.8934
CC	26.36	1	26.36	4.38	0.1045
CD	52.56	1	52.56	8.73	0.0417 *
DD	1.81	1	1.81	0.30	0.6125
Lack-of-fit	212.62	10	21.26	3.53	0.1176
Pure error	24.07	4	6.02		
Total (corr.)	3422.55	28			
R^2^	0.9308				
Adj R^2^	0.8617				
**DPPH**					
A	460.04	1	460.04	189.86	0.0002 *
B	72.52	1	72.52	29.93	0.0054 *
C	6.31	1	6.31	2.60	0.1819
D	65.80	1	65.80	27.16	0.0065 *
AA	20.94	1	20.94	8.64	0.0424 *
AB	1.44	1	1.44	0.59	0.4838
AC	3.06	1	3.06	1.26	0.3238
AD	3.61	1	3.61	1.49	0.2893
BB	40.43	1	40.43	16.69	0.0150 *
BC	63.20	1	63.20	26.08	0.0069 *
BD	4.00	1	4.00	1.65	0.2682
CC	22.88	1	22.88	9.44	0.0372 *
CD	1.32	1	1.32	0.55	0.5010
DD	1.99	1	1.99	0.82	0.4165
Lack-of-fit	108.49	10	10.85	4.48	0.0808
Pure error	9.69	4	2.42		
Total (corr.)	902.51	28			
R^2^	0.8691				
Adj R^2^	0.7381				
**FRAP**					
A	2892.31	1	2892.31	499.79	0.0000 *
B	0.00	1	0.00	0.00	0.9910
C	61.65	1	61.65	10.65	0.0310 *
D	13.23	1	13.23	2.29	0.2051
AA	0.71	1	0.71	0.12	0.7432
AB	1.10	1	1.10	0.19	0.6850
AC	91.20	1	91.20	15.76	0.0165 *
AD	55.50	1	55.50	9.59	0.0363 *
BB	28.42	1	28.42	4.91	0.0910
BC	42.90	1	42.90	7.41	0.0528
BD	15.60	1	15.60	2.70	0.1759
CC	22.66	1	22.66	3.92	0.1189
CD	10.89	1	10.89	1.88	0.2420
DD	56.67	1	56.67	9.79	0.0352 *
Lack-of-fit	214.98	10	21.50	3.71	0.1088
Pure error	23.15	4	5.79		
Total (corr.)	3548.47	28			
R^2^	0.9329				
Adj R^2^	0.8658				
**aloin**					
A	696.16	1	696.16	186.04	0.0002 *
B	0.21	1	0.21	0.06	0.8230
C	0.03	1	0.03	0.01	0.9330
D	0.48	1	0.48	0.13	0.7383
AA	11.79	1	11.79	3.15	0.1505
AB	5.76	1	5.76	1.54	0.2825
AC	10.24	1	10.24	2.74	0.1734
AD	1.21	1	1.21	0.32	0.6000
BB	8.55	1	8.55	2.29	0.2051
BC	3.61	1	3.61	0.96	0.3816
BD	3.24	1	3.24	0.87	0.4048
CC	7.87	1	7.87	2.10	0.2205
CD	0.25	1	0.25	0.07	0.8088
DD	4.67	1	4.67	1.25	0.3266
Lack-of-fit	37.29	10	3.73	1.00	0.5501
Pure error	14.97	4	3.74		
Total (corr.)	809.15	28			
R^2^	0.9354				
Adj R^2^	0.8708				

A: ethanol concentration; B: extraction temperature; C: extraction time; D: solvent volume. * significant effect at *p* < 0.05.

**Table 4:** Single response optimized extraction conditions and predicted values.

Response	Et (%)	T (°C)	t (min)	V (mL)	Predicted Value
Yield	40.0	67.7	26.7	80.0	26.8 g AVE 100 g AVS^−1^
TPC	80.0	40.0	5.0	56.0	127.4 mg GAE g AVE^−1^
DPPH	80.0	80.0	40.0	52.7	73.4 mg TE gAVE^−1^
FRAP	80.0	54.4	39.9	80.0	140.5 mg TE gAVE^−1^
aloin_MAX_	80.0	40.0	29.4	80.0	59.0 mg gAVE^−1^
aloin_MIN_	40.0	40.3	40.0	61.8	35.4 mg gAVE^−1^

Et: ethanol concentration; T: extraction temperature; t: extraction time; V: solvent volume.

**Table 5:** Phenolic compounds identified in AVE by HPLC-MS, analytical figures of merit and quantification results by HPLC-DAD.

Peak ^1^	Compound	(*m*/*z*) [M-H]^-^	t_R_ (min)	Calibration Range (mg kg^−1^)	Linearity (R^2^)	LOD (mg kg^−1^)	LOQ (mg kg^−1^)	RSD ^2^ (%)	AVE (mg 100 gAVS^−1^)
1	aloesin	393	9.3	0.06–61.80	0.9998	0.164	0.546	2.6	292.6 ± 0.5
2	chlorogenic acid	353	10.5	0.05–99.70	0.9960	0.213	0.711	1.3	80.0 ± 0.2
3	orientin	447	14.4	0.01–11.20	0.9991	0.061	0.203	2.3	46.5 ± 0.1
4	aloeresin D	555	19.1	0.05–9.82	0.9971	0.383	1.275	2.6	39.7 ± 1.1
5	aloin B	417	19.2	0.10–100.60	0.9998	0.087	0.292	1.1	308.1 ± 0.6
6	aloin A	417	20.0	0.20–202.10	0.9999	0.278	0.926	0.9	702.0 ± 2.0
7	cinnamic acid	147	25.2	0.004–3.700	0.9992	0.029	0.095	1.4	13.6 ± 0.5
8	aloe emodin	269	29.4	0.001–0.900	0.9961	0.018	0.061	1.8	3.6 ± 0.1

^1^ peak assignation corresponding to Figure 4. ^2^ within-day precision (*n* = 3 at all concentration levels used in the calibration range).

## Data Availability

Data are contained within the article.
